# Galvanic corrosion inhibition from aspect of bonding orbital theory in Cu/Ru barrier CMP

**DOI:** 10.1038/s41598-021-00689-6

**Published:** 2021-10-27

**Authors:** Kangchun Lee, Seho Sun, Ganggyu Lee, Gyeonghui Yoon, Donghyeok Kim, Junha Hwang, Hojin Jeong, Taeseup Song, Ungyu Paik

**Affiliations:** 1 Department of Energy Engineering, Hanyang University, Seoul, South Korea; 2 Department of Nanoscale Semiconductor Engineering, Hanyang University, Seoul, South Korea

**Keywords:** Surface chemistry, Electronic devices

## Abstract

In this report, the galvanic corrosion inhibition between Cu and Ru metal films is studied, based on bonding orbital theory, using pyridinecarboxylic acid groups which show different affinities depending on the electron configuration of each metal resulting from a π-backbonding. The sp^2^ carbon atoms adjacent to nitrogen in the pyridine ring provide π-acceptor which forms a complex with filled d-orbital of native oxides on Cu and Ru metal film. The difference in the d-orbital electron density of each metal oxide leads to different π-backbonding strength, resulting in dense or sparse adsorption on native metal oxides. The dense adsorption layer is formed on native Cu oxide film due to the full-filled d-orbital electrons, which effectively suppresses anodic reaction in Cu film. On the other hand, only a sparse adsorption layer is formed on native Ru oxide due to its relatively weak affinity between partially filled d-orbital and pyridine groups. The adsorption behaviour is investigated through interfacial interaction analysis and electrochemical interaction evaluation. Based on this finding, the galvanic corrosion behaviour between Cu and Ru during chemical mechanical planarization (CMP) processing has been controlled.

## Introduction

The contact of two metals with different electrical potentials in an electrolyte induces galvanic corrosion^1,2^. Especially in the semiconductor manufacturing process, the galvanic corrosion issues are constantly being pointed out as the various metals such as copper (Cu), tantalum (Ta), aluminium (Al), tungsten (W), cobalt (Co), and ruthenium (Ru) are used in interconnect, a barrier layer (liner), via filling and bottom electrode applications depending on the technology node developed. The device structure composed of these metals is constructed through chemical mechanical planarization (CMP) process followed by thin film deposition via chemical vapour deposition (CVD) and/or electroplating deposition (EPD)^3,4^.

**Figure 1:**
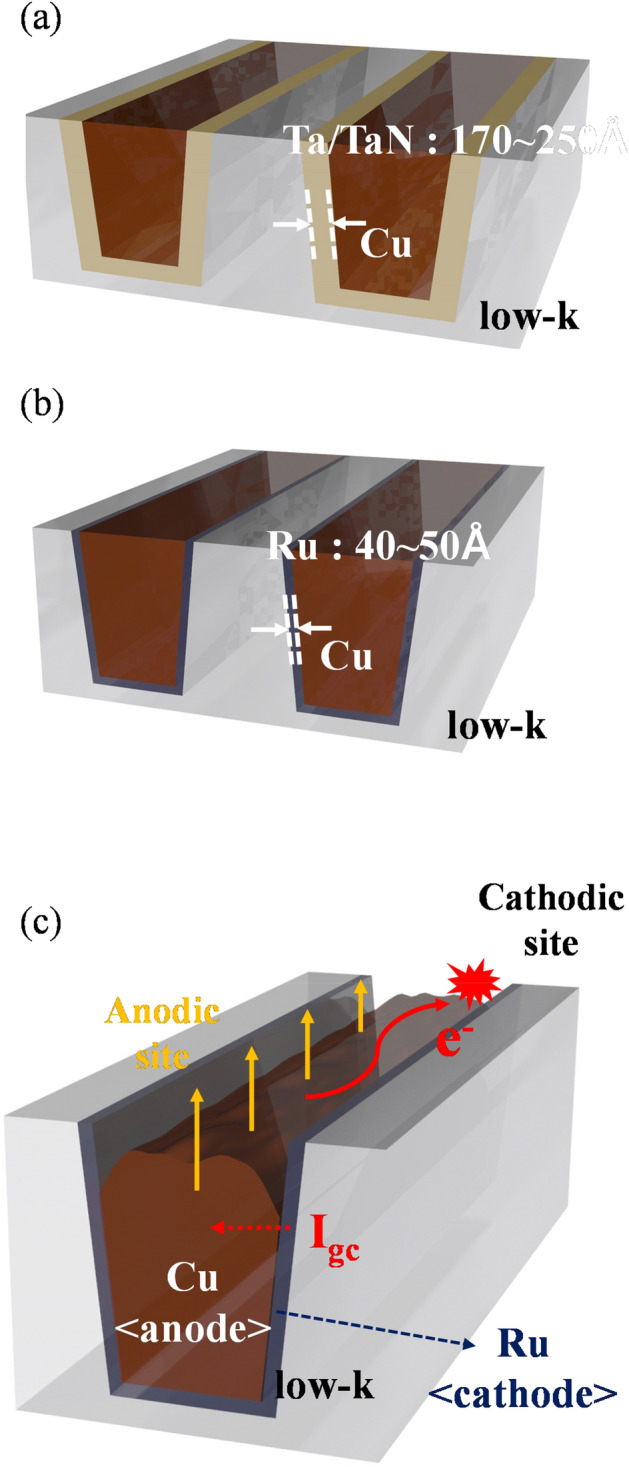
Schematic illustration of **(a)** Ta/TaN barrier structure, **(b)** Ru barrier structure, and **(c)** galvanic corrosion in a Cu/Ru bimetallic system; for Cu interconnect in back-end-of-the-line (BEOL) process.

As the device feature size shrinks to 5 nm and smaller, the Ta/TaN material, used as the diffusion barrier metal layer with a thickness of 170–250 Å in Cu interconnects, reaches a limit in the achievement of a complete Cu gap-fill and prevent electromigration (EM) of Cu (Fig. 1a)^5–8^. To overcome these limitations, Ru has been attracted much attention as a barrier layer material due to its excellent conductivity and gap-fill property. By employing Ru as a barrier layer material, the thickness of barrier metal could be reduced to 40–50 Å, resulting in decreased resistance–capacitance delay (Fig. 1b). It also meets integrated circuit (IC) design rule requirements of 5 nm and smaller. Furthermore, due to the compatibility between Cu and Ru metals, Cu could be deposited directly onto the Ru film via electroplating without requiring a seed layer, which is advantageous in terms of cost reduction and surface quality of the deposited film^9–11^. For the application of the Ru metal in barrier structure, the CMP performance is required to 1:1 selectivity of removal rate on Ru and Cu metal films.

Ru is a chemically stable material and relatively hard compared to Cu (Ru: Mohs 6.5 and Cu: Mohs 2.5). For the chemical activation of the Ru metal, potassium periodate (KIO_4_) and hydrogen peroxide (H_2_O_2_) are used as auxiliary oxidants in CMP slurries^12,13^. However, during the CMP process, excessive use of these oxidants brings galvanic corrosion deteriorating between the Cu and Ru films (Fig. 1c), resulting in defects that will degrade the device performances (i.e., RC delay deterioration) consequently. In Ru barrier structure in Cu interconnects, Cu film becomes the anode (oxidation reaction as an electron donor), and Ru film acts as the cathode (reduction reaction as an electron acceptor) due to the electromotive force difference between Cu and Ru films^14,15^. Research and engineering reactions at the interface between metal films and electrolytes are crucial for reducing the electromotive force difference, especially interface resistance controlling the galvanic corrosion. In recent studies, Peethala et al. reported inhibitor substances in specific KIO_4_ concentrations to understand how to prevent Cu and Ru galvanic corrosion. The benzotriazole (BTA) and ascorbic acid (AA) can help minimize the galvanic corrosion of Cu with preferential absorption of AA on Ru, which suppressed the cathodic reaction at Ru^16^. Also, Chen et al. studied that the periodate ions also formed a complex with Cu^17^. The reaction between periodate ions and dissolved Cu ions forming the Cu(IO_3_)_2_ ∙ nH_2_O is the accelerative stage of the galvanic corrosion of the Cu/Ru couple in KIO_4_ solution^18^. They suggest that adsorbed ions and chemicals act as a passivation film synergistically. At the presence of K_2_MoO_4_ and benzotriazole (BTA), adsorbed MoO_4_^2-^ ions on the metal films increase activation energy of the corrosion and form a three-dimensional network complex film with the BTAs due to the ion–dipole effect of absorbed MoO_4_^2-^ ions^19^. However, there is much room for research on selecting inhibitors and engineering the slurries regarding the inhibition mechanism of Cu/Ru galvanic corrosion.

Herein, we proposed the design principle of inhibitors to minimize the galvanic corrosion at Cu/Ru coupled films based on bonding orbital theory. The d-orbital electron densities that distinguish between native metal oxides of the Cu and Ru metal films cause selective adsorption affinity of the pyridine groups by π-back bonding, resulting in reduced electromotive force differences. For the analysis of selective adsorption behavior of the pyridine groups, the contact angle measurements and X-ray photoelectron spectroscopy (XPS) were conducted depending on the concentration of the pyridine group, nicotinic acid. The difference in affinity for each metal of the nicotinic acid showed a dissimilarity in the density of the inhibitor layer formation. Also, the electromotive force differences were evaluated by Tafel slope and electrochemical impedance spectroscopy (EIS) measurement. The open-circuit potential (ΔE_oc_) difference between Cu and Ru films is 0.49 V conducted at a 0.05 M KIO_4_ solution with 3% H_2_O_2_ at pH 10.0. A dense layer was formed on the Cu film in the presence of nicotinic acid, leading to the potential gap reduction between the two films from 0.49 to 0.09 V. Finally, the change in polishing removal rate and Cu to Ru selectivity were calculated through the CMP test.

## Experimental section

### Materials preparation

Commercially colloidal SiO_2_ was used as an abrasive (d_mean_ ~ 70 nm, Fuso, Japan). The solid concentration of SiO_2_ was 5.0 wt%. 3.0 wt% hydrogen peroxide (H_2_O_2_, Junsei Chemical, Japan) was used as an oxidant. Nicotinic acid (Sigma Aldrich, USA) was added to the solution per solid concentration as an inhibitor. 0.01 M manganese(II) nitrate hydrate (Sigma Aldrich, USA) as a catalyst and 0.1 M of citric acid (Sigma Aldrich, USA) as a chelating agent were used. 0.05 M potassium periodate (Sigma Aldrich, USA) was used as an auxiliary oxidizer. The slurry pH was adjusted to 10.0 using potassium hydroxide (KOH, 1.0 N, Daejung Chemical, Korea). 300 mm electroplating Cu wafers were purchased from Advantech Korea Co., Ltd. The ruthenium wafers made through chemical vapor deposition were supplied by MEMC Korea Ltd.

### Adsorption behavior analysis

The adsorption behavior of inhibitor onto the Cu and Ru films was analyzed through contact angle measurements (Digidrop, GBX, Ireland) and X-ray photoelectron spectroscopy (XPS) (K-Alpha + , Thermo Fisher Scientific Messtechnik, USA). The Cu and Ru films were individually dipped in 200 mL of various inhibitor concentrations at pH 10.0 for 10 min. Then samples were subsequently rinsed with deionized water before analysis.

### Electrochemical performance investigation

Potentiodynamic polarization measurement (AUT320N, Metrohm AUTOLAB, Switzerland) and electrochemical impedance spectroscopy (EIS) (AUT320N, Metrohm AUTOLAB, Switzerland) were used to characterize the electrochemical performance of Cu and Ru films. The counter electrode was a platinum-coated mesh, and the reference electrode was an Ag/AgCl containing 3 M KCl electrolyte. The size of cut samples for potentiodynamic polarization measurement and electrochemical impedance spectroscopy were 3 × 6 cm (exposing 1 cm^2^ active area) and 1 × 3 cm (exposing 1 cm^2^ active area), respectively (All the samples prepared in this measurement was the same as that mentioned in 2.2.). Before each experiment, the Cu and Ru wafer were removed from the native oxide using a buffered oxide etch (BOE) solution (Sigma-Aldrich, USA). The EIS was measured in a frequency range from 10^5^ Hz to 10^–2^ Hz, and the applied AC potential was 1 × 10^–2^ V_rms_ in amplitude.

### CMP performance evaluation

A coupon CMP (POLI-300, G&P Technology, Korea) with a pad (IC 1010/Suba IV, Rohm, and Haas Electronic Materials, USA) was used for the CMP evaluation. The thickness of copper and ruthenium films was measured using a Four-Point Probe (FFP) (CMT-SR5000, Changmin Tech, Korea). The specific resistance (ρ) of Cu and Ru was 1.68 × 10^–8^ Ω·m and 7.1 × 10^–8^ Ω·m, respectively, and the thickness was calculated by dividing by the sheet resistance estimated through FFP in a constant condition. The CMP evaluation to obtain material removal rates of both Cu and Ru films was performed three times under the 1.5 psi pressure, 79/80 rpm rotation speed, and 100 mL/min flow rate conditions.

## Results and discussion

### Inhibition mechanism of pyridine functional group of nicotinic acid

**Figure 2:**
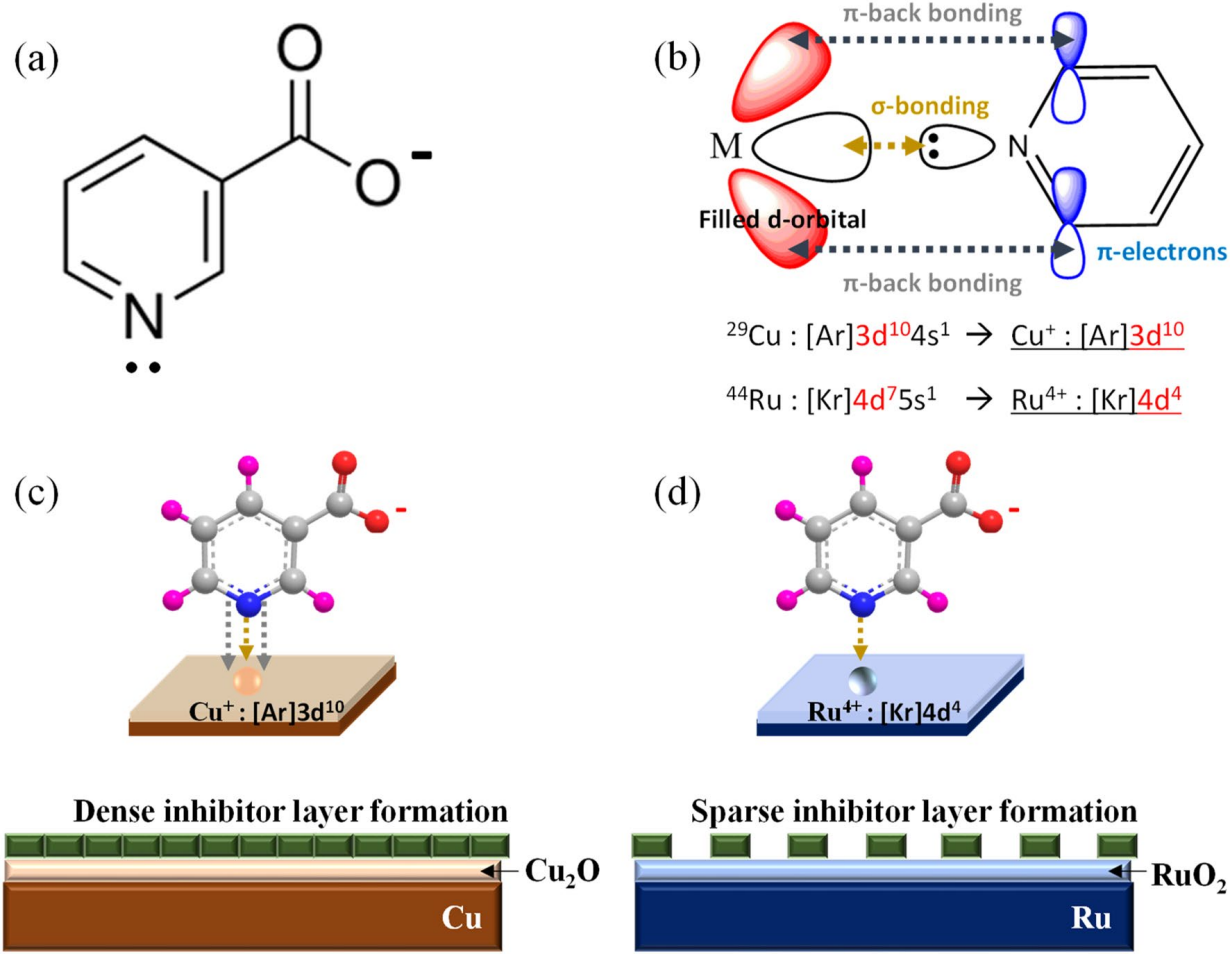
**(a)** Structure of nicotinic acid. **(b)** Formation of σ-bonding and π-back bonding between pyridine functional group and metal ion. The inhibition mechanism of nicotinic acid on **(c)** Cu blanket wafer and **(d)** Ru blanket wafer.

Figure 2 represents the structure of 3-pyridinecarboxylic acid (nicotinic acid) and the schematic illustration of its suggested inhibition mechanism. As shown in Fig. 2a, nicotinic acid has the formula C_6_H_5_NO_2_ and belongs to a group of monocarboxaylic derivatives of pyridine. Since the nitrogen atom of the pyridine functional group has electron pairs, they can form complexes by using σ-bonding (i.e., coordinate covalent bonding) with transition metal ions from oxidized metal surfaces such as Cu, Ir, W, Co, and Ru^20^. Such complexes mentioned above form a passivation layer on the metal film surface, preventing corrosion reactions by preventing water adsorption. However, the density of the inhibitor layer through the metal complex differs depending on the type of metal film. As shown in Fig. 2b, the electron configurations of metal ions in oxidized Cu and Ru films are Cu^+^: (Ar)3d^10^ and Ru^4+^: (Kr)4d^4^, respectively. As a result, there is a difference in terms of π-back bonding which participates in the delocalized π-electrons present in planar cyclic hydrocarbon molecules of the pyridine ring structure and filled d-orbital of metal ions (Fig. 2b)^21^. In the case of Cu film, the d-orbital of Cu^+^ ion derived from Cu_2_O is fully occupied. Therefore, the π-electron of pyridine ring structure can form π-backbonding with the Cu^+^ ion better than the Ru^4+^ ion because of the insufficient outermost electrons of the Ru^4+^ ion. Consequently, the affinity between nicotinic acid and Cu film should be more robust than its affinity with Ru film, resulting in a dense inhibitor layer formation on the surface of Cu oxide (Fig. 2c). In comparison, only a sparse inhibitor layer is formed on the Ru oxide film (Fig. 2d).

### Interfacial interaction behavior analysis between Cu/Ru films and nicotinic acid inhibitor

**Figure 3:**
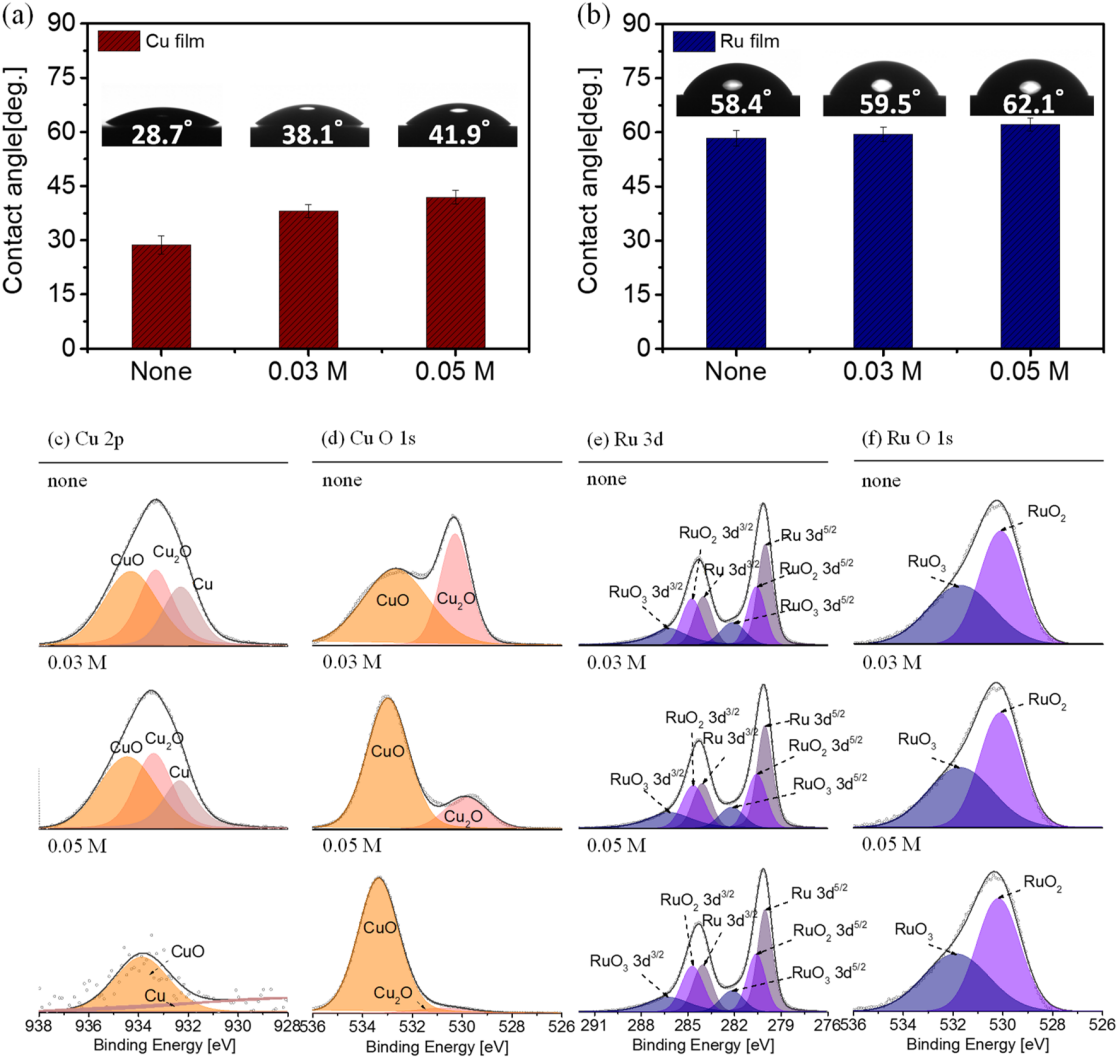
Interfacial interaction behaviour analysis between Cu/Ru films and nicotinic acid inhibitor. **(a,b)** Contact angle measurement of Cu and Ru films after dipping as a function of inhibitor concentrations at pH 10. **(c–f)** X-ray photoelectron spectroscopy (XPS) results for **(c)** Cu 2p, **(d)** Cu O1s, **(e)** Ru 3d, and **(f)** Ru O1s as a function of inhibitor concentrations at pH 10.

For analysis of interfacial interaction between Cu/Ru films and nicotinic acid, the contact angle measurement and X-ray photoelectron spectroscopy (XPS) are evaluated. The contact angle measurements are a valuable tool for measuring the hydrophobicity of thin films. In general, the nitrogen atom from the pyridine functional group of nicotinic acid forms a complex by creating a σ-bond between its electron pair and metal ions from oxidized metal films. It forms an adsorption layer and makes a hydrophobic surface^22^. The contact angle values of Cu and Ru films as a function of nicotinic acid concentrations were shown in Fig. 3a,b. The contact angle values of Cu and Ru immersed in a slurry without nicotinic acid were 28.7° and 58.4°, respectively. The difference in the contact angle value means that the intrinsic surface property of Ru film is more hydrophobic than the Cu film. With the addition of nicotinic acid, the contact angle values of Cu and Ru films increased to 41.9° and 62.1°, respectively. The increase of contact angle values (i.e., increased hydrophobicity) of both Cu and Ru films with nicotinic acid-treated is commonly attributed to a specific orientation of adsorbed nicotinic acid molecules hydrophobic pyridine group to form a protective hydrophobic layer. The change in contact angle value with and without nicotinic acid was 13.2° for Cu and 3.7° for Ru, and the amount of contact angle change is large for Cu film. It means that nicotinic acid adsorbed more onto the Cu film than the Ru film.

The XPS analysis was utilized to characterize the chemical composition changes of Cu and Ru films after being immersed for 10 min in different slurries (Fig. 3c–f). Figure 3c,d show the exemplary spectra of Cu film; Cu 2p and Cu O1s, respectively. In Fig. 3c, the metallic Cu binding energy for 2p^3/2^ is located at 932.2 eV. The Cu binding energies for CuO and Cu_2_O are found at 933.8 eV and 932.0 eV, respectively. These results are consistent with the other research reports^23–26^. The Cu and Cu_2_O peak intensities were significantly decreased according to the concentration of the nicotinic acid. At the addition of the 0.03 M nicotinic acid, there was no significant change in Cu 2p peak intensity. However, it was confirmed that when the concentration was increased to 0.05 M, the Cu 2p peak intensity was overall lowered. The decrease in Cu 2p peak intensity is that nicotinic acid adsorbed onto the Cu_2_O, forming a dense inhibitor layer (Fig. S1).

Meanwhile, the binding energy peaks for CuO and Cu_2_O in the Cu O1s spectra are detected at 534.0 eV and 529.7 eV, respectively. The Cu O1s peak from Fig. 3d also shows that the Cu_2_O peak rapidly decreases with the nicotinic acid addition. This suggests that the pyridine functional group of nicotinic acid adsorbs well to Cu_2_O (Cu^+^ state, fully occupied d-orbital) rather than CuO (Cu^2+^ state, partially occupied d-orbital).

Figure 3e,f show the fine spectra of Ru film; Ru 3d and Ru O1s, respectively. The metallic Ru binding energies for 3d^5/2^ and 3d^3/2^ are located at 280.0 eV and 284.4 eV^27,28^. Ru binding energy for RuO_2_ is found to be 280.8 eV and 285.0 eV. Because RuO_2_∙H_2_O is a metallic oxide with a partially filled conduction band, the core–hole coupling may occur on this surface^28^. Thus, RuO_3_ is considered present with the bulk phase of RuO_2,_ and the binding energy peaks at 282.3 eV and 286.5 eV, respectively. Meanwhile, the binding energy peaks for RuO_2_ and RuO_3_ appear at 529.2 eV and 530.7 eV in Ru O1s spectra^29,30^. No change with nicotinic acid concentration was observed in the case of both Ru 3d (Fig. 3e) and Ru O1s (Fig. 3f) XPS results. In other words, the above results mean that nicotinic acid was barely adsorbed on the Ru film. These results correspond with the contact angle measurements.

### Electrochemical interaction evaluation between Cu/Ru films and nicotinic acid inhibitor

**Figure 4:**
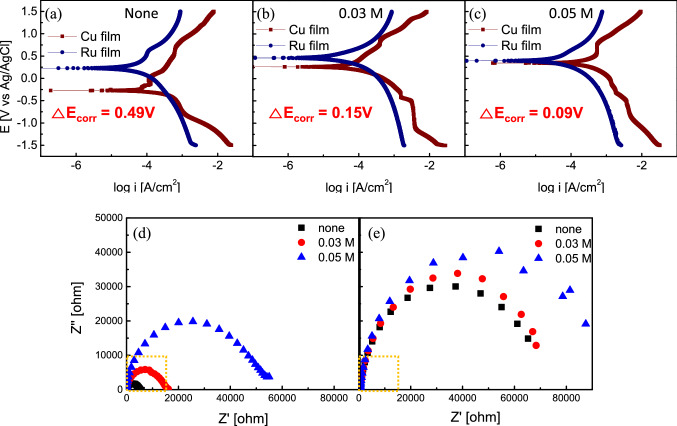
Electrochemical interaction evaluation between Cu/Ru films and nicotinic acid inhibitor. Potentiodynamic plots for Cu and Ru in solution as a function of inhibitor concentrations at pH 10 **(a–c)**: **(a)** w/o nicotinic acid, **(b)** 0.03 M nicotinic acid, and **(c)** 0.05 M nicotinic acid. Electrochemical impedance spectroscopy (EIS) data are showing Nyquist plots for **(d)** Cu film and **(e)** Ru film in solution for various inhibitor concentrations at pH 10.

For analysis on electrochemical properties, the potentiodynamic plots and Nyquist plots are evaluated. The corrosion potentials (E_corr_) and corrosion currents (I_corr_) of the Cu and Ru films under the different nicotinic acid concentrations are noted in Fig. 4a–c and Table 1. As shown in Fig. 4a–c, the Tafel curves of Cu and Ru films were evaluated through potentiodynamic polarization measurements. The potential difference between Cu and Ru films decreased from 0.49 to 0.09 V as inhibitor concentrations increased from 0 to 0.05 M. The galvanic corrosion occurs in the heterojunction of Cu/Ru films because two metal films have different potentials. In Cu film, oxidation reactions that donate electrons occur (as an anodic site), while reduction reactions occur in the Ru film that accepts electrons (as a cathodic site). Therefore, by controlling the potential of both films by reducing the gap of potential difference, the redox reaction can be suppressed, resulting in galvanic corrosion prevention. The experimental results in Fig. 4c show that the potential difference between Cu and Ru is significantly reduced to 0.09 V, indicating the galvanic corrosion between Cu and Ru films was suppressed. On the other hand, the Cu film potential change is 0.63 V (from −0.27 V to 0.36 V), more significant than that of Ru film (0.23 V, from 0.22 V to 0.45 V). In both cases, adsorption of nicotinic acid tended to form an inhibitor passivation layer leading to the potential value increase. Still, the E_corr_ value change of Cu film was more extensive than that of Ru film. This phenomenon is due to the denser layer formation on the Cu film, consistent with the adsorption affinity trends of nicotinic acid mentioned above. Electrochemical impedance spectroscopy (EIS) was performed to evaluate the barrier protection properties of nicotinic acid to both Cu and Ru films. The impedance data were fitted using the electrical equivalent circuits with three resistances and two constant phase elements (CPE) shown in Fig. S2. R_s_ represents the solution resistance, and R_f_ is the film resistance. R_1_ includes the R_ct_ (charge transfer resistance), R_d_ (diffusion layer resistance), and R_a_ (accumulation resistance) at the metal/solution interface^31,32^. The CPE_1_ and CPE_2_ represent the film capacitance and electric double-layer capacitance, respectively. From Fig. S2, the R_p_ (polarization resistance) values, representing the corrosion inhibition effect characteristics, consisted of R_f_ and R_1_. Therefore, obtaining a high R_p_ value indicates an improved inhibition effect. In Table 2, the R_p_ value change is more considerable for Cu film because nicotinic acid adsorbed more onto Cu film. That is consistent with the adsorption behaviours and potentiodynamic polarization measurements described earlier.

**Table 1:** The corrosion potentials (E_corr_) and corrosion currents (I_corr_) value of each Cu and Ru film according to inhibitor concentration.

Solution system	Cu film		Ru film	
	E_corr_ (V)	I_corr_ (mA/cm^2^)	E_corr_ (V)	I_corr_ (mA/cm^2^)
None	−0.27	0.22	0.22	0.05
Nicotinic acid 0.03 M	0.26	0.34	0.41	0.31
Nicotinic acid 0.05 M	0.36	0.47	0.45	0.42

**Table 2:** Polarization resistance (R_p_) and inhibition efficiency (η) of metal films according to inhibitor concentration.

Solution system	Cu film		Ru film	
	R_p_ (Ω∙cm^2^)	η (%)	R_p_ (Ω∙cm^2^)	η (%)
None	5704.2	–	69,518	–
Nicotinic acid 0.03 M	13,830	58.75	74,011	6.07
Nicotinic acid 0.05 M	49,897	88.57	78,549	11.50

Nyquist plots as a function of nicotinic acid concentrations for Cu and Ru films at pH 10 are shown in Fig. 4d,e, respectively. As nicotinic acid concentrations increase, the real impedance difference at lower and higher frequencies for both films was increased, leading to an increase in R_p_ value (Table 2). That is owing to the formation of the inhibitor protection layer on each film surface. Detailed impedance parameters and inhibition efficiencies (η %) are listed in Table 2. The inhibition efficiency could be calculated from the polarization resistance values as follows:

$$\upeta \left[\mathrm{\%}\right]= \frac{{R}_{p}-{R}_{p}^{0}}{{R}_{p}}\times 100$$

(Where, ${R}_{p}^{0}$ is the polarization resistance without nicotinic acid.) The increase in the inhibition effect (i.e., increasing R_p_ value) due to nicotinic acid adsorption is more dramatic in Cu film (Fig. 4d and Fig. S3) comparing with Ru film (Fig. 4e and Fig. S4).

### Effect of pyridine functional group on Cu and Ru removal rate and surface roughness

**Figure 5:**
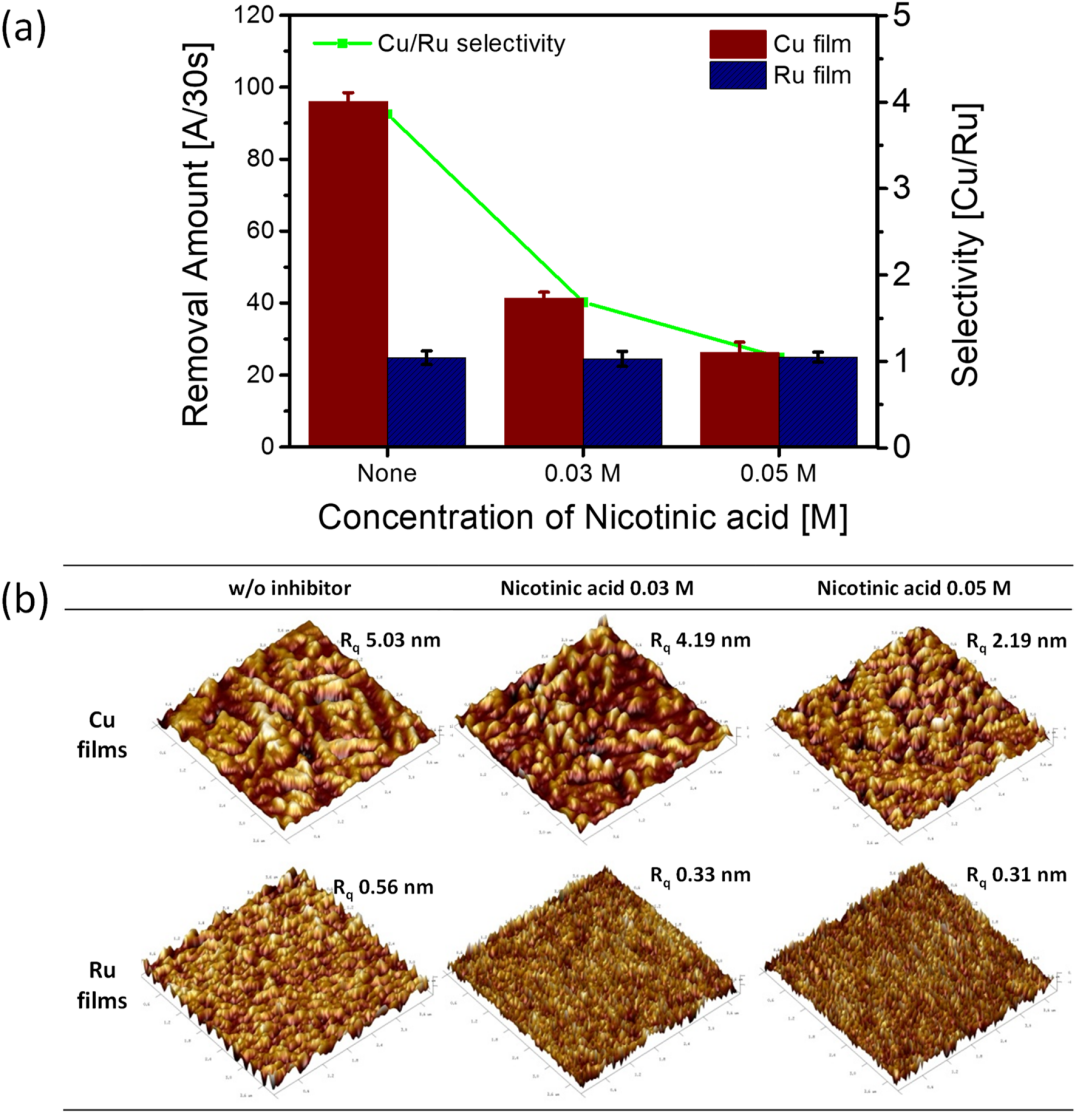
CMP performance for Cu and Ru films as a function of inhibitor concentrations at pH 10. **(a)** The removal rate of the metal film via nicotinic acid concentration. **(b)** The surface morphologies of metal films on the surface under different nicotinic acid concentrations through AFM measurement.

Figure 5a and Table 3 represent the removal rates of Cu and Ru films under the different inhibitor conditions and their removal selectivity at pH 10 by the CMP process. Before the CMP process, the colloidal stability of each slurry is observed by zeta potential analysis (Fig. S5) and large particle counter evaluation (Fig. S6), which shows no harmful effects on the colloidal stability as CMP slurries. Without nicotinic acid as an inhibitor, the initial removal rates of Cu and Ru film were 95.98 Å/30 s and 24.85 Å/30 s, respectively, with a Cu to Ru selectivity of 3.86. However, as nicotinic acid content increased, the removal rate of Cu film decreased steeply from 95.98 Å/30 s to 26.23 Å/30 s. In contrast, the removal rate of Ru film was maintained at a constant value within the margin of error range regardless of the nicotinic acid concentration (Ultimately optimized with the Cu to Ru selectivity of 1:1). The affinity between nicotinic acid and Ru film is relatively smaller than that of Cu film, confirmed by XPS and contact angle results above. Therefore, the amount of nicotinic acid adsorbed on Ru film is comparatively weak and insignificant. This result is consistent with the small potential change (ΔE_corr_: 0.23 V) observed by the potentiodynamic polarization measurements. In a sub-5 nm logic semiconductor device using a ruthenium barrier structure, the Cu to Ru selectivity requirement to achieve a completely flat surface is 1:1 for the Ru barrier CMP^12,19^. That is to minimize defects such as dishing, erosion and protrusion. Therefore, using nicotinic acid as an inhibitor with an affinity difference between Cu and Ru films prevents galvanic corrosion and controls the Cu to Ru selectivity simultaneously.

**Table 3:** Results of the removal rate and selectivity between Cu/Ru films according to inhibitor concentration.

	Cu removal rate (Å/30 s)	Ru removal rate (Å/30 s)	Selectivity (Cu/Ru)
None	95.98	24.85	3.86
Nicotinic acid 0.03 M	41.33	24.52	1.69
Nicotinic acid 0.05 M	26.23	25.0	1.05

Additionally, as shown in Fig. 5b, the root-mean-square (RMS) value of surface roughness (R_q_) was estimated using atomic force microscope (AFM) for each sample of Cu and Ru. Since Ru is a chemically more inert material compared to Cu, the R_q_ value of Ru is smaller than that of Cu in all cases regardless of the nicotinic acid concentration. Meanwhile, as the concentration of nicotinic acid increased, the R_q_ value of Cu gradually decreased. This indicates that a smooth surface with improved roughness was obtained because the dissolution rate of Cu film was suppressed by forming a dense inhibiting layer. On the other hand, the improvement of Ru roughness is much smaller than that of the Cu film due to the suggested sparse inhibiting layer formation on Ru film.

## Conclusions

The pyridine functional group inhibiting mechanism of nicotinic acid for Cu and Ru films was thoroughly investigated. Based on the results from adsorption affinity experiments (i.e., contact angle measurement and XPS) and electrochemical performances (i.e., potentiodynamic polarization measurement and EIS), we came to the following conclusions:

1. The difference in adsorption affinity of the pyridine functional group for Cu and Ru films is attributed to the difference in the electron configuration between Cu^+^ ions and Ru^4+^ ions.
2. Nicotinic acid with a pyridine ring structure has a better affinity for Cu film than Ru film because it forms both σ-bonds and π-back bonds.
3. Therefore, a denser inhibitor layer is formed on the copper oxide from Cu film, which reduces the anodic reactions.
4. The increased electrical potential in Cu film due to the dense inhibitor layer can significantly reduce its potential gap compared to Ru film. That leads to suppressing galvanic corrosion for Cu / Ru couples.
5. Furthermore, the dense inhibitor layer reduces the Cu removal rate by forming a passivation bed. As a result, almost 1:1 selectivity for copper to ruthenium could be achieved in the Ru barrier structure during the CMP process.

## Supplementary Information

Supplementary Figures.
