# Supplementary material for: Galvanic corrosion inhibition from aspect of bonding orbital theory in Cu/Ru barrier CMP
Source: Sci Rep. 2021 Oct 27;11:21214. doi: 10.1038/s41598-021-00689-6 (PMC8551296; doi:10.1038/s41598-021-00689-6)
Supplement: Supplementary file 1 — Supplementary Figures. [file 41598_2021_689_MOESM1_ESM.docx]

**sGalvanic Corrosion Inhibition from Aspect of Bonding Orbital Theory in Cu/Ru barrier CMP**

Kangchun Lee^1, =^, Seho Sun^1, =^, Ganggyu Lee^1^, Gyeonghui Yoon^2^, Donghyeok Kim^2^,

Junha Hwang^1^, Hojin Jeong^2^, Taeseup Song^1, z^, and Ungyu Paik^1, 2, z^

*^1^Department of Energy Engineering, Hanyang University, Seoul, South Korea*

*^2^Department of Nanoscale Semiconductor Engineering, Hanyang University, Seoul, South Korea*

^z^Co corresponding author: Professor Ungyu Paik (upaik@hanyang.ac.kr)

Professor Taeseup Song (tssong@hanyang.ac.kr)

Tel. +82-2-2220-0502

Fax +82-2-2281-0502

**Supplementary figures**

**Figure S1.** X-ray photoelectron spectroscopy result for C1s of Cu film as a function of inhibitor concentration at pH 10.0.

**
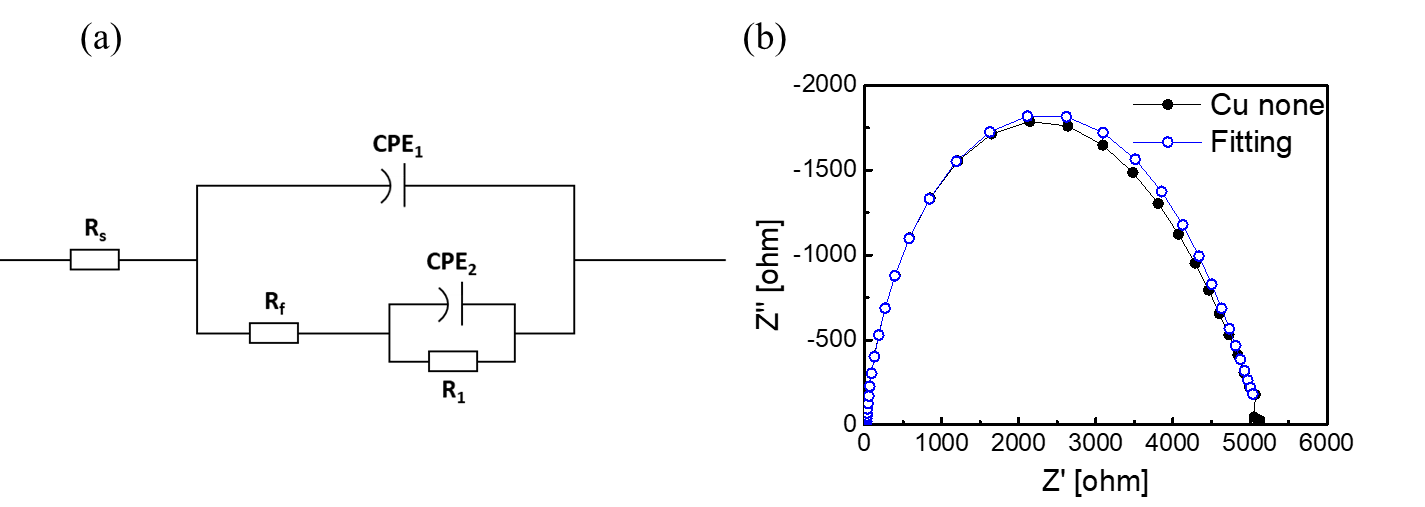
**

**Figure S2**. Equivalent circuits of the impedance spectra obtained in different inhibitor concentrations at pH 10 for Cu film and Ru film. (a) equivalent circuit model. (b) fitted curves of Nyquist plot of Cu film without inhibitor.

**Figure S3**. Magnified graph of Nyquist plot of Cu film according to nicotinic acid concentration at pH 10.0.

**Figure S4**. Magnified graph of Nyquist plot of Ru film according to nicotinic acid concentration at pH 10.0.


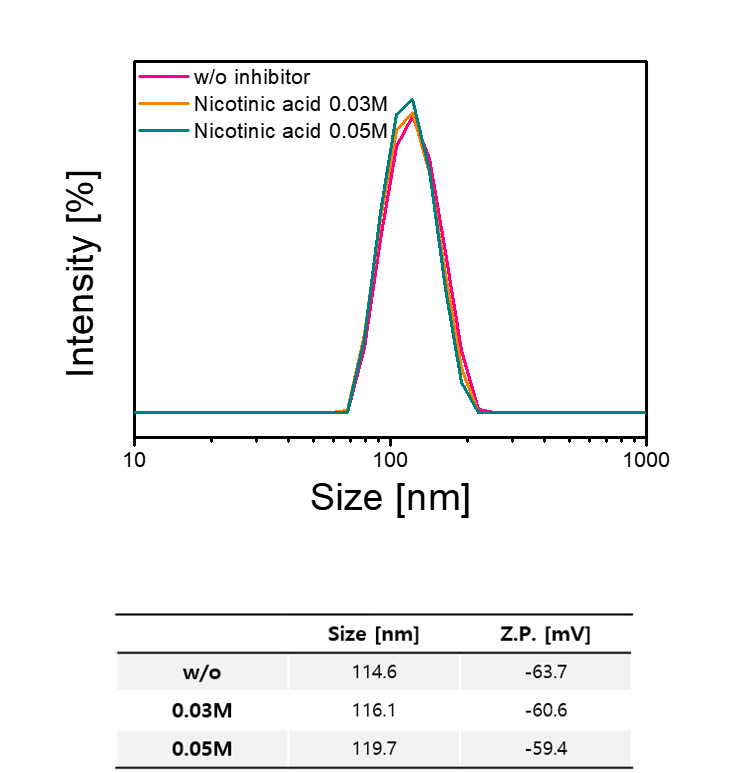


**Figure S5**. Zeta sizer and zeta potential evaluation for dispersion stability of CMP slurry according to concentration of nicotinic acid


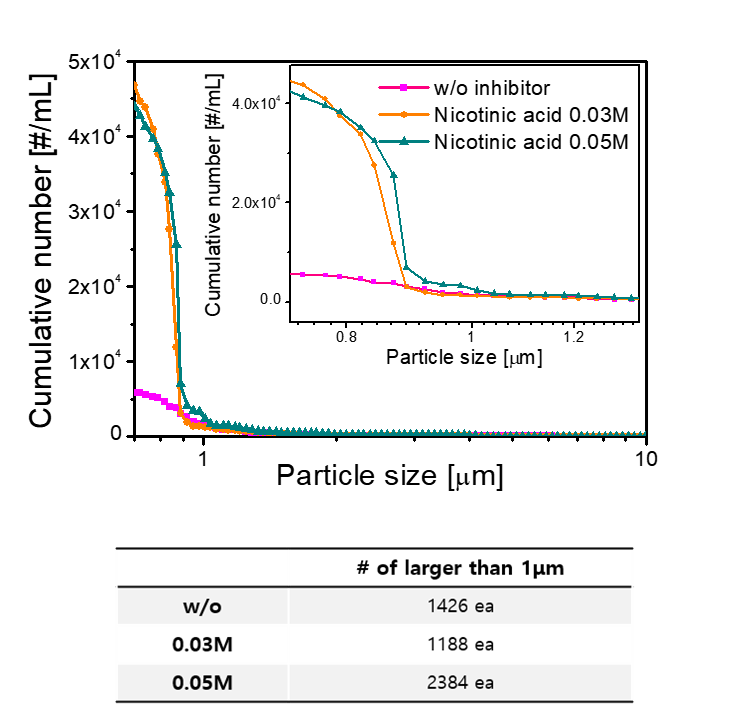


**Figure S6**. Large particle counter evaluation for dispersion stability of CMP slurry according to concentration of nicotinic acid
